# Supplementary material for: EnzML: multi-label prediction of enzyme classes using InterPro signatures
Source: BMC Bioinformatics. 2012 Apr 25;13:61. doi: 10.1186/1471-2105-13-61 (PMC3483700; doi:10.1186/1471-2105-13-61)
Supplement: Addtional file 5 — The Java code to format the data files, evaluate and predict. The file enzml_java_code.tar.gz contains the Java code used to format database data to ARFF and XML formats, to execute cross and train-test (jackknife) evaluations and to record evaluation results to database. More information is included in the readme.txt file and the Javadoc files. The code can be used with a MySQL database. To use a different database software, other JDBC drivers might be required. [file 1471-2105-13-61-S5.gz › java_code/enzml2011/doc/uk/ac/ed/inf/csb/taweka/class-use/DataSetFactory.html]

Uses of Class uk.ac.ed.inf.csb.taweka.DataSetFactory


---


|  |  |  |
| --- | --- | --- |
| **Package** \| **Class** \| **Use** \| **Tree** \| **Deprecated** \| **Index** \| **Help** | |  |
| PREV   NEXT | **FRAMES**    **NO FRAMES**     **All Classes** |  |


---


## **Uses of Class uk.ac.ed.inf.csb.taweka.DataSetFactory**


Uses of DataSetFactory in uk.ac.ed.inf.csb.taweka

| Methods in uk.ac.ed.inf.csb.taweka that return DataSetFactory | |
| --- | --- |
| `DataSetFactory` | `InstancesFactory.getDataSet()` |

| Methods in uk.ac.ed.inf.csb.taweka with parameters of type DataSetFactory | |
| --- | --- |
| `static void` | `ArffFileWriter.writeArffFile(DataSetFactory dataSetFactory, java.lang.String filePathAndName)` |

Constructors in uk.ac.ed.inf.csb.taweka with parameters of type DataSetFactory

`InstancesFactory(DataSetFactory dataSet)`

---


|  |  |  |
| --- | --- | --- |
| **Package** \| **Class** \| **Use** \| **Tree** \| **Deprecated** \| **Index** \| **Help** | |  |
| PREV   NEXT | **FRAMES**    **NO FRAMES**     **All Classes** |  |


---
